# Lenalidomide regulates the CCL21/CCR7/ERK1/2 axis to inhibit migration and proliferation in diffuse large B-cell lymphoma

**DOI:** 10.32604/or.2024.050036

**Published:** 2024-12-20

**Authors:** WEN YANG, BIN TANG, DAN XU, WENXIU YANG

**Affiliations:** Department of Pathology, Guizhou Medical University, The Affiliated Hospital of Guizhou Medical University, Guiyang, 550004, China

**Keywords:** CCR7, CCL21, ERK1/2, Lenalidomide, Diffuse large B-cell lymphoma (DLBCL)

## Abstract

**Background:**

The prognostic significance of the chemokine receptor CCR7 in diffuse large B-cell lymphoma (DLBCL) has been reported previously. However, the detailed mechanisms of CCR7 in DLBCL, particularly regarding its interaction with lenalidomide treatment, are not fully understood.

**Methods:**

Our study utilized bioinformatics approaches to identify hub genes in SU-DHL-2 cell lines treated with lenalidomide compared to control groups. Immunohistochemical data and clinical information from 122 patients with DLBCL were analyzed to assess the correlation of CCR7 and p-ERK1/2 expression with the prognosis of DLBCL. Furthermore, *in vitro* and *in vivo* experiments were conducted to clarify the role of CCR7 in the response of DLBCL to lenalidomide treatment.

**Results:**

Our bioinformatics analysis pinpointed CCR7 as a hub gene in the context of lenalidomide treatment in DLBCL. Notably, 31.14% and 36.0% (44/122) of DLBCL cases showed positive expression for CCR7 and ERK1/2 respectively, establishing them as independent prognostic factors for adverse outcomes in DLBCL via multivariate Cox regression analysis. Additionally, our studies demonstrated that the external application of the protein CCL21 promoted proliferation, migration, invasion, and activation of the ERK1/2 pathway in SU-DHL-2 and OCI-LY3 cell lines with high levels of CCR7 expression. This effect was mitigated by CCR7 silencing through siRNA, application of ERK inhibitors, or lenalidomide treatment. *In vivo* experiments reinforced the efficacy of lenalidomide, significantly reducing tumor growth rate, tumor mass, serum total LDH levels, and expression of CCR7 and p-ERK1/2 in a SU-DHL-2 xenograft model in nude mice (*p* < 0.05).

**Conclusion:**

Our study clarifies the potential role of the CCL21/CCR7/ERK1/2 axis in the therapeutic effects of lenalidomide in DLBCL treatment.

## Introduction

Diffuse large B-cell lymphoma (DLBCL), the most common type of non-hodgkin lymphoma, accounts for 25%–45% of all new lymphoma diagnoses annually [1]. The standard first-line treatment for DLBCL involves a combination of chemotherapy and immunotherapy, specifically the retuximab, cyclophosphamide, doxorubicin, vincristine, and prednisone (R-CHOP) regimen, which achieves a cure rate of 60% to 70% [2]. Despite this, about 30% to 40% of patients face relapse within two years following treatment, including 10% who relapse within six months or fail to respond significantly to the initial treatment [3]. For these patients, treatment with lenalidomide, either as monotherapy or in combination with R-CHOP, has demonstrated clinical efficacy, particularly in the non-germinal center B-cell (GCB) subtype of DLBCL where lenalidomide shows a higher response rate [4,5]. However, lenalidomide’s effectiveness shows considerable variability in clinical trials [6,7], highlighting the critical need for biomarkers capable of forecasting lenalidomide’s treatment responses. Identifying these biomarkers is crucial for guiding clinical decision-making and understanding the mechanisms behind lenalidomide resistance in DLBCL.

In the pathophysiology of DLBCL, chemokine receptors and their ligands play a pivotal role [8]. Previous research has indicated that lenalidomide potentially modulates CCR7 and CCL21 [9,10]. Furthermore, the role of CCR7 and CCL21 in DLBCL has been documented. For instance, a study involving 61 patients with primary nodal DLBCL revealed higher expression of CCR7 in the non-GCB subtype compared to the GCB subtype, with its elevated expression correlating with poorer overall survival rates, thereby establishing it as an independent prognostic factor in the disease [11]. The ligand CCL21, by recruiting and activating Treg cells, may similarly impact the prognosis of DLBCL patients [12]. However, research into chemokine receptors and their ligands, especially CCR7/CCL21, in the context of lenalidomide resistance in DLBCL, remains limited. In light of this, we have employed a comprehensive approach, including *in vitro* and *in vivo* experimental models, bioinformatics analysis, and clinical samples, to assess the function of CCR7/CCL21 in DLBCL, particularly regarding their role in lenalidomide resistance mechanisms. This study aims to enhance our understanding of DLBCL treatment mechanisms, specifically concerning lenalidomide resistance, thereby providing a scientific basis for future therapeutic strategies.

## Materials and Methods

### Human samples

This study included 122 adult patients with DLBCL who were treated at the Affiliated Hospital of Guizhou Medical University between 2017 and 2022. The inclusion criteria were: histopathologically confirmed diagnosis; initial diagnosis of primary cancer; and availability of comprehensive clinical and follow-up data. Exclusion criteria included: history of malignant tumors, transplantation, or immunodeficiency, and incomplete data. The GCB subtype was identified according to the world health organization classification scheme [13]. This research received approval from the Ethics Committee of the Affiliated Hospital of Guizhou Medical University, under the approval numbers 595. Publication of this study was conducted with informed consent from all participants. All research methodologies adhered to the standards set forth in the Declaration of Helsinki.

### Cell culture

The DLBCL cell lines SU-DHL-2, OCI-Ly3, and U2932 were sourced from the National Collection of Authenticated Cell Cultures, Shanghai, China. Following authentication via short tandem repeat (STR) profiling, these cell lines were cultured in RPMI 1640 medium supplied by Bio-Channel Biotechnology Co. Ltd., Nanjing, China, enriched with 10% fetal bovine serum from Gibco, NY, USA, and 1% penicillin/streptomycin from Beyotime, Shanghai, China. The culture environment was maintained at 37°C in an atmosphere containing 5% CO_2_. Lenalidomide was procured from Selleck, USA; the MEK inhibitor U0126 (Catalog #9903) was obtained from CST, USA; and human recombinant CCL21 protein (Product Code: 10447-HNAB) was acquired from Sino Biological, Beijing, China.

### Animals

Six-week-old female BALB/c nude mice (18–20 g) from Ziyuan Animal Technology Co., Ltd. (Hangzhou, China) acclimatized for one week in a controlled barrier facility. The mice were housed in a controlled barrier facility with a 12-hour light/dark cycle, temperature maintained at 22 ± 2°C, and humidity at 50 ± 10%, with free access to sterilized food and water. All housing and care conditions were in accordance with the ARRIVE guidelines to ensure ethical standards. For the study initiation, 2 × 10^7^ SU-DHL-2 cells in 200 µL PBS were subcutaneously injected into the mice’s right axillary region. Upon tumors reaching 5 mm in diameter, mice were allocated into control (n = 5) and Lenalidomide-treated groups (n = 5), receiving 100 mg/kg Lenalidomide or 5% DMSO vehicle respectively via intraperitoneal injection every two days for a total of eight doses. Tumor measurements were taken bi-daily, calculating volume as 1/2 (Length × Width^2^). Post-treatment, mice were euthanized for cardiac blood sampling and serum LDH levels were assessed using an Abbott Architect ci1600-2 analyzer and LDH kits (Roche, Germany). Tumors were fixed in 10% formalin, processed, and paraffin-embedded for HE staining and IHC antigen detection. This study received ethical approval from the Affiliated Hospital of Guizhou Medical University Ethics Committee, under the approval numbers 2304785.

### Small interfering RNA (siRNA) transfection

The siRNA targeting CCR7 (si-CCR7) and a negative control siRNA (si-NC) were acquired from Sangon Biotech, Shanghai, China. The sequences for si-CCR7 were: sense 5′-CGUGUUGACCUAUAUCUAUUUTT-3′and antisense 5′-AAAUAGAUAUAGGUCAACACGTT-3′. The SU-DHL-2 cell line underwent transfection with si-CCR7 or si-NC using RNAtransmate (Catalog No. E607402, Sangon Biotech, Shanghai, China), following the provided protocol. For each group, 2 × 10^5^ cells were transfected with 100 nM siRNA, plated in 6-well plates, and incubated at 37°C in a 5% CO_2_ atmosphere. Knockdown efficiency was assessed by western blot 72 h post-transfection.

### Cell counting kit (CCK)-8

To evaluate cell viability, the Cell Counting Kit-8 (CCK-8; Catalog #MA0218, Meilunbio, Dalian, China) was utilized as per the manufacturer’s guidelines. Cells were plated in 96-well plates at a concentration of 5,000 cells per well and underwent various treatments. Subsequently, CCK-8 assays were performed at designated time points to assess cell viability. The absorbance of the resulting solution was measured at 450 nm using the EnSpire Multilabel Plate Reader (Varioskan LUX, Thermo Fisher Scientific, USA), employing absorbance spectrometry to determine the optical density.

### EdU assay

After treatment, cells were plated at a density of 2 × 10^5^ cells per well in 6-well plates and then exposed to 30 mM EdU (RiboBio, Guangzhou, China) for 3 h. Following incubation, cells underwent fixation with 4% paraformaldehyde, treatment with 2 mg/mL glycine, and permeabilization with 0.5% Triton X-100. After permeabilization, cells were washed with PBS and stained with Apollo solution for EdU detection, followed by a PBS wash to remove excess stain. Nuclei were then stained with Hoechst 33342 solution for 30 min to visualize DNA. After final washes with PBS, images of the stained cells were captured from four randomly selected fields using a fluorescence microscope.

### RNA sequencing

SU-DHL-2 cells underwent lenalidomide treatment at a concentration of 90 µM, while control cells were treated with an identical concentration of Dimethyl Sulfoxide (DMSO) for a duration of 72 h. The RNA extraction, library preparation, and sequencing processes adhered to previously established methods [14]. Specifically, total RNA was extracted in accordance with the instructions provided by Novogene Bioinformatics Technology Co. Ltd., China. The sequencing was conducted at Novogene, Tianjin, China, using the Illumina Novaseq platform to generate 150 bp paired-end reads.

### IHC staining assay

IHC staining was performed as previously detailed [15]. The evaluation of IHC staining targeted CCR7 (monoclonal antibody, 1:500; ab253187, Abcam, UK) and p-ERK 1/2 (monoclonal antibody, 1:400; #4370, CST, USA), utilizing a semi-quantitative scoring system that measures staining intensity within tumor cells as detailed in reference [16]. Positive expression for CCR7 and p-ERK1/2 was determined based on thresholds established in previous research [11,17].

### Immunofluorescence staining

Polychromatic immunofluorescence staining was performed utilizing the four-color multiple fluorescent immunohistochemical staining kit (abs50012, Absin, Shanghai, China), in accordance with the manufacturer’s protocol. The primary antibodies applied included CCR7 (1:500) and p-ERK (1:400), consistent with those utilized in immunohistochemical analyses, alongside CD20 (1:500, Zhongshan, Beijing, China).

### Western blotting

Total protein was extracted and quantified using the bicinchoninic acid (BCA) assay, followed by separation via sodium dodecyl sulfate-polyacrylamide gel electrophoresis (SDS-PAGE). Proteins were then transferred to polyvinylidene fluoride (PVDF) membranes and incubated overnight at 4°C with primary antibodiess: anti-GAPDH (1:2000; #ab9485, Abcam), anti-tubulin (1:5000; #ab6160, Abcam), anti-CCR7 (1:1000; #ab191575, Abcam), anti-p ERK1/2 (1:1000; #ab278538, Abcam), and anti-ERK1/2 (1:1000; #ab17942, Abcam). After washing, membranes were incubated with HRP-conjugated secondary antibody (1:2000;#ab288151, Abcam) for 1 hour at room temperature. Bands were detected using enhanced chemiluminescence (ECL) and imaged with the ChemiDocTM MP System (BIO-RAD). Band intensities were analyzed using ImageJ software, with the target protein expression normalized to GAPDH or tubulin levels.

### Transwell assay

Migration and invasion assays were performed using transwell chambers with an 8 μm pore size (Corning Costar, NY, USA). For invasion, 100 μL of Matrigel was applied to the upper chamber to establish a gel barrier. We seeded 1.25 × 10^5^ cells in 200 μL of serum-free medium into the upper chamber, experimenting with additions such as CCL21 human recombinant protein (1 μg/mL), U0126 (10 μM), and lenalidomide. The lower chamber was filled with 600 μL of serum-free medium, with the inclusion of CCL21 (1 μg/mL) to act as a chemoattractant. After 24 to 48 h, cells that migrated or invaded through the pores to the lower chamber were stained using a suitable method, such as crystal violet staining. The stained cells were then quantified by manual counting under a microscope to assess the migration and invasion capabilities.

### Quantitative reverse transcription–polymerase chain reaction (qRT‒PCR)

Total RNA was purified following previously described methods and reverse-transcribed using a cDNA reverse transcription kit (Tiangen, Beijing, China). Real-time PCR was then performed using a Bio-Rad iQ5 real-time PCR system. Primer sequences are provided in the Table A1.

### Bioinformatics analysis

Differential expression genes (DEGs) between control and lenalidomide-treated groups was determined using a false discovery rate (FDR) < 0.05 and |log_2_ FC| ≥ 1, via the limma R package (version 3.54.0). Gene ontology (GO) and Kyoto encyclopedia of genes and genomes (KEGG) pathway enrichment analyses were then performed on identified genes, applying a threshold of corrected *p*-values < 0.05. The top 15 hub genes were obtained using the maximal clique centrality (MCC) algorithm through the cytoHubba plugin in cytoscape (version 3.6.1).

### Statistical analysis

All experiments were conducted in triplicate, with data analysis and graphical presentations carried out using SPSS software (version 21.0) and GraphPad Prism (version 6). Comparisons of means ± standard deviations were performed using independent *t*-tests, while chi-square tests were utilized for comparisons of proportions. Survival analysis was conducted using log-rank tests, and Cox regression analysis was applied to evaluate the prognostic significance of various factors in patients with DLBCL. Statistical significance for all analyses was set at *p* < 0.05.

## Results

### Differential gene expression and pathway analysis in lenalidomide-treated DLBCL

**Figure 1:**
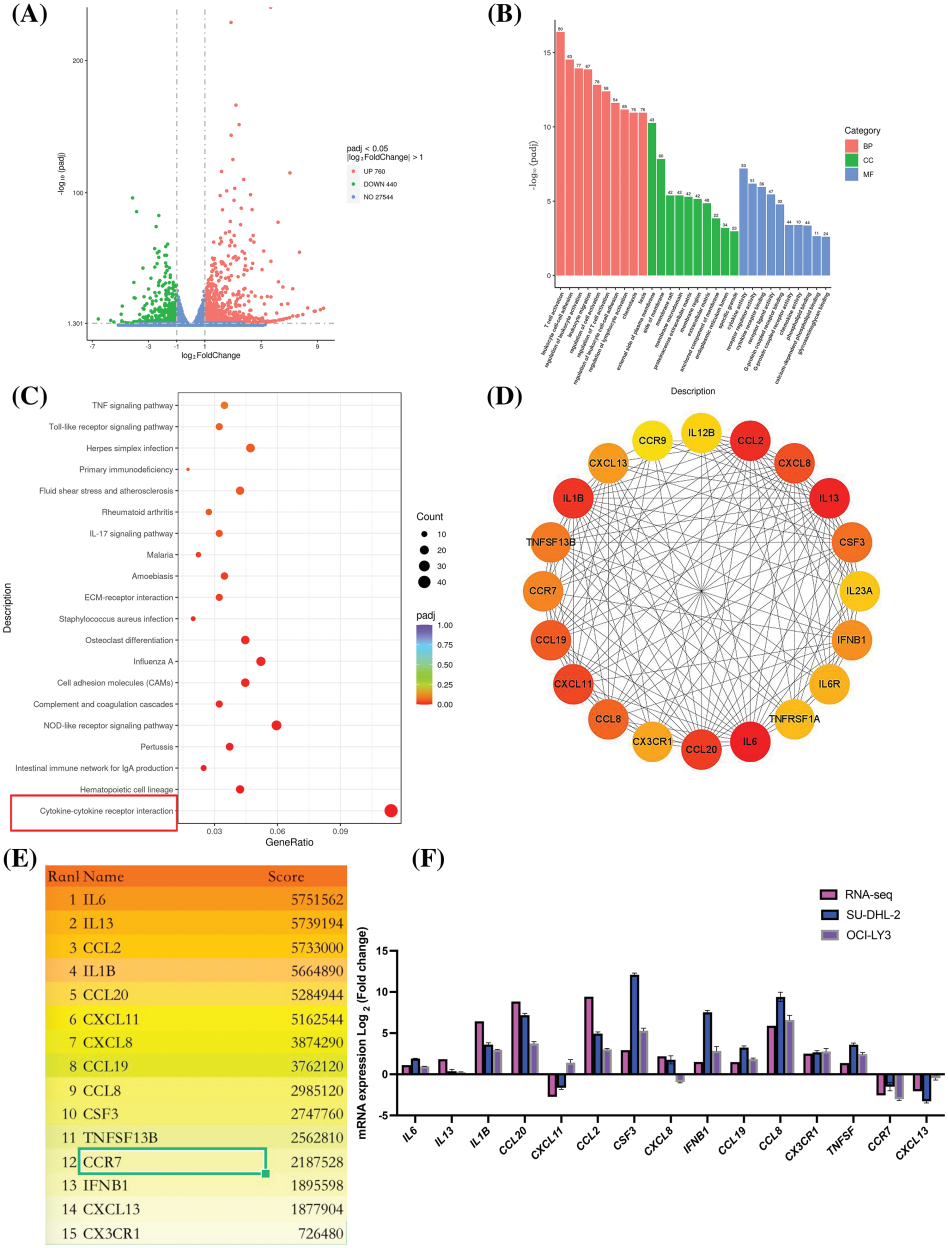
Analysis of DEGs and pathways in DLBCL cell lines treated with lenalidomide. (A) Volcano plot illustrating the distribution of DEGs. (B) Top 10 GO terms identified in BP, CC, and MF. (C) Top 20 pathways highlighted by KEGG enrichment analysis. (D) Protein interaction network from DEGs in the cytokine-cytokine receptor interaction pathway. (E) Ranking of the top 15 hub genes based on MCC scores. (F) Validation of RNA-seq findings through qRT-PCR analysis.

Through RNA sequencing analysis, we identified 1,200 DEGs between lenalidomide-treated groups and control groups, including 760 upregulated and 440 downregulated genes (Fig. 1A). GO enrichment analysis revealed the top 10 most significant GO terms in the categories of biological processes (BP), cellular components (CC), and molecular functions (MF) (Fig. 1B). Specifically, BP involved T-cell activation, leukocyte cell-cell adhesion, and regulation of leukocyte activation; CC enrichment was mainly observed in the external side of the plasma membrane and membrane raft; while MF predominantly involved cytokine activity and cytokine receptor binding. KEGG pathway analysis highlighted significant enrichment in cytokine-cytokine receptor interaction pathways (Fig. 1C), underscoring the pivotal role of cytokines in the impact of Lenalidomide on DLBCL. To further obtain hub genes in lenalidomide’s effect on DLBCL, we utilized the MCC algorithm to select the top 15 core genes (Figs. 1D and 1E). Notably, CCR7, as the leading chemokine receptor (Fig. 1E), was chosen for subsequent research focus. Additionally, to verify the reliability of the RNA-Seq data, qRT-PCR analysis was conducted on 15 selected genes, comparing fold changes to RNA-Seq expression profiles. The high consistency between qRT-PCR and mRNA-seq results, as shown in Fig. 1F, further confirmed the accuracy of the transcriptome sequencing data.

### CCR7 expression decreased with increasing in lenalidomide concentration in DLBCL cell lines

**Figure 2:**
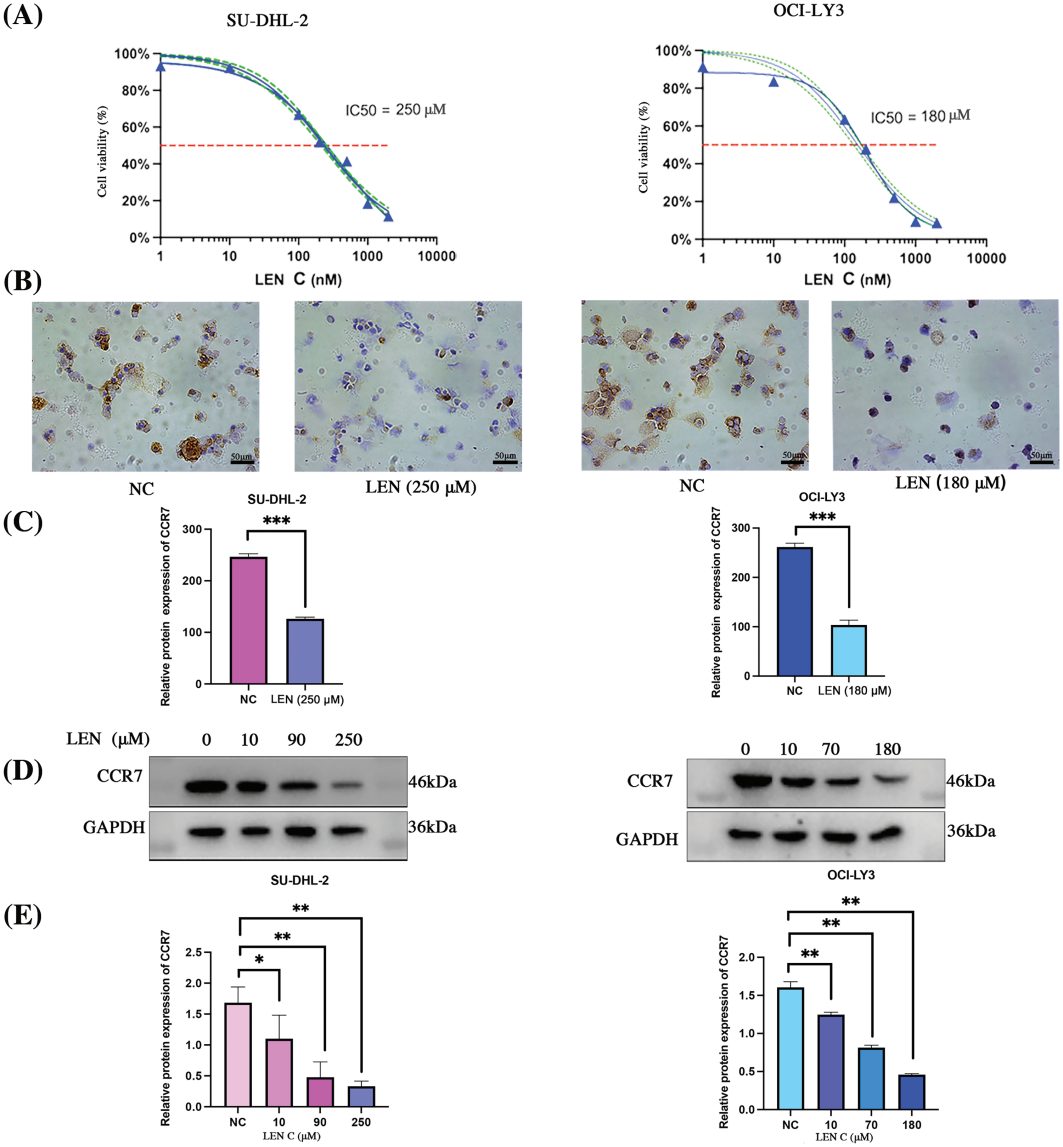
Impact of lenalidomide concentration on CCR7 expression in DLBCL cell lines. (A) Dose-response curves for lenalidomide on SU-DHL-2 and OCI-LY3 cells over 72 h. (B) IHC analysis showing reduced CCR7 expression in cells treated with lenalidomide. (C) Bar chart indicating the IHC scores for CCR7 expression across different lenalidomide concentrations. (D) WB analysis demonstrating a dose-dependent decrease in CCR7 expression with increasing lenalidomide concentrations. (E) Graphical representation of CCR7’s relative expression levels (*p < 0.05, **p < 0.01, ***p < 0.001).

The dose-response effect of lenalidomide on SU-DHL-2 and OCI-LY3 cells over 72 h was illustrated in Fig. 2A. Cells were subsequently exposed to varying lenalidomide concentrations (IC_10_, IC_30_ and IC_50_) for 72 h, using an equivalent dose of DMSO (10 µM) as the control. IHC and WB analyses revealed a dose-dependent decrease in CCR7 expression levels with increasing concentrations of lenalidomide (Figs. 2B–2E).

### CCR7 and p-ERK1/2 as independent prognostic risk factors in DLBCL

**Figure 3:**
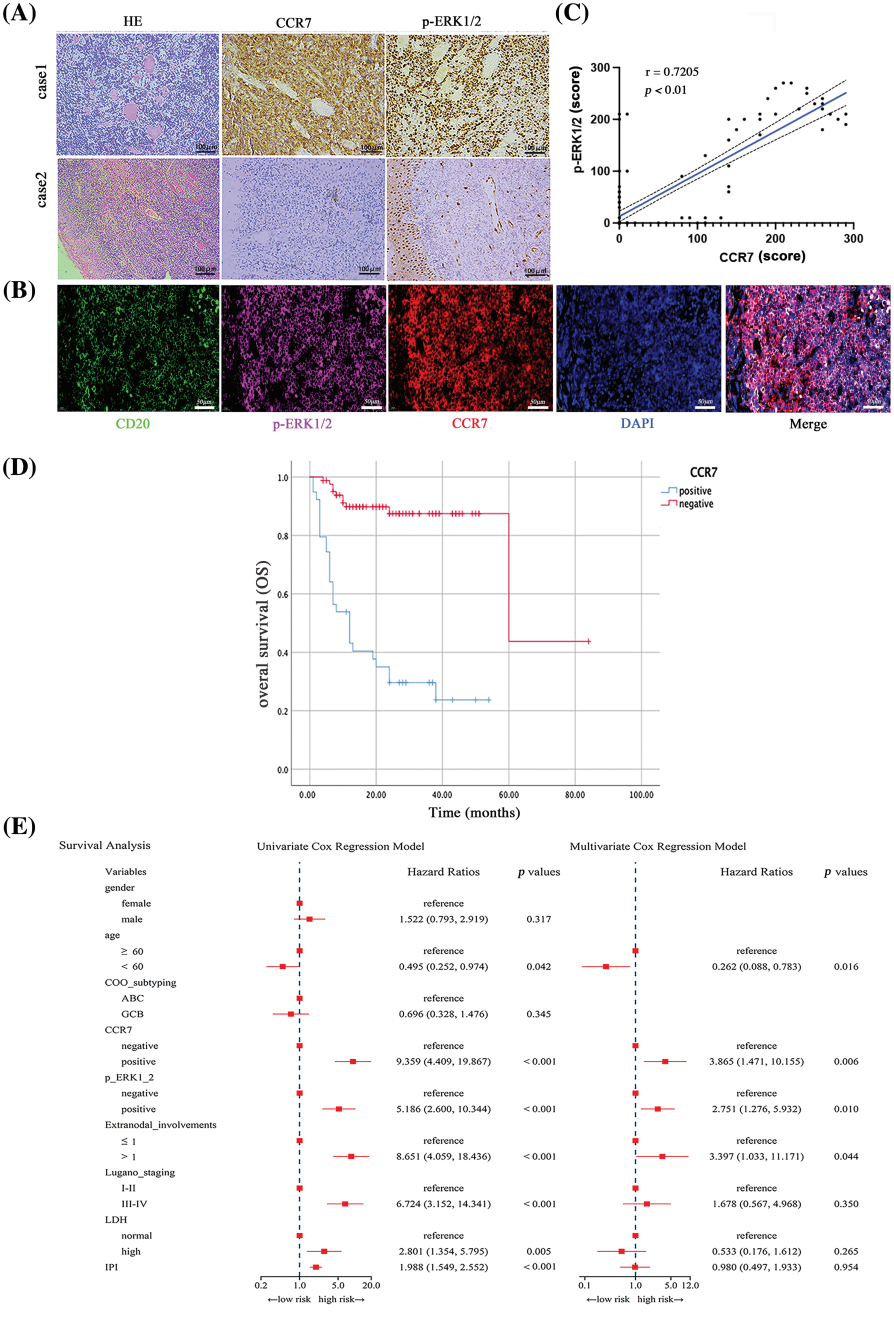
The impact of CCR7 and p-ERK1/2 expression on prognosis in DLBCL patients. (A) Representative i IHC images of positive and negative expressions of CCR7 and p-ERK1/2 in DLBCL tissue samples (×200). (B) Multicolor immunofluorescence staining illustrating the co-localization of CD20, p-ERK1/2, and CCR7 in DLBCL (×200). (C) Scatter plot demonstrating the correlation between CCR7 and p-ERK1/2 expression levels. (D) Kaplan-Meier survival curves comparing the outcomes of patients with positive *vs*. negative CCR7 expression. (E) Forest plots from univariate and multivariate Cox regression analyses assessing the prognostic risk associated with DLBCL patients.

Considering the potential roles of CCR7 in DLBCL, we utilized clinical data to delve deeper into its specific functions within DLBCL. Among 122 clinical samples of DLBCL, CCR7 expression was positively identified in 38 cases (31.14%). As detailed in Table A2, a strong positive correlation emerged between CCR7 expression and higher Lugano stages, elevated international prognostic index (IPI) scores, increased lactate dehydrogenase (LDH) levels, multiple lesion involvements, and cell of origin (COO), particularly marking a higher positivity rate in the non-GCB subtype (*p* < 0.05). However, no significant correlation was observed between CCR7 expression and other parameters such as age or gender (*p* > 0.05). Given the close association between CCR7 and p-ERK1/2, we also assessed the p-ERK1/2 levels in these cases (Fig. 3A), revealing a positive correlation between the two proteins (*p* < 0.05) (Fig. 3B). Table A3 displayed the relationship between p-ERK1/2 expression and clinical parameters, showing differences from CCR7, with p-ERK1/2 positively correlated with advanced age (≥60 years), the presence of multiple involvement sites, higher Lugano stages, and elevated IPI scores, but not significantly associated with gender or COO. Immunofluorescence demonstrated the co-localization of p-ERK1/2, CCR7, and CD20 in DLBCL tumor cells (Fig. 3C). Kaplan-Meier survival analysis indicated that patients with positive CCR7 expression faced poorer overall survival outcomes (Fig. 3D). Through univariate and multivariate Cox regression analyses, CCR7 and p-ERK 1/2 expressions were identified as significant predictors of adverse survival outcomes (Fig. 3E). In summary, CCR7 and p-ERK 1/2 serve as independent risk factors in DLBCL, potentially playing a pivotal role.

### CCL21/CCR7 interaction and its oncogenic role in DLBCL progression

**Figure 4:**
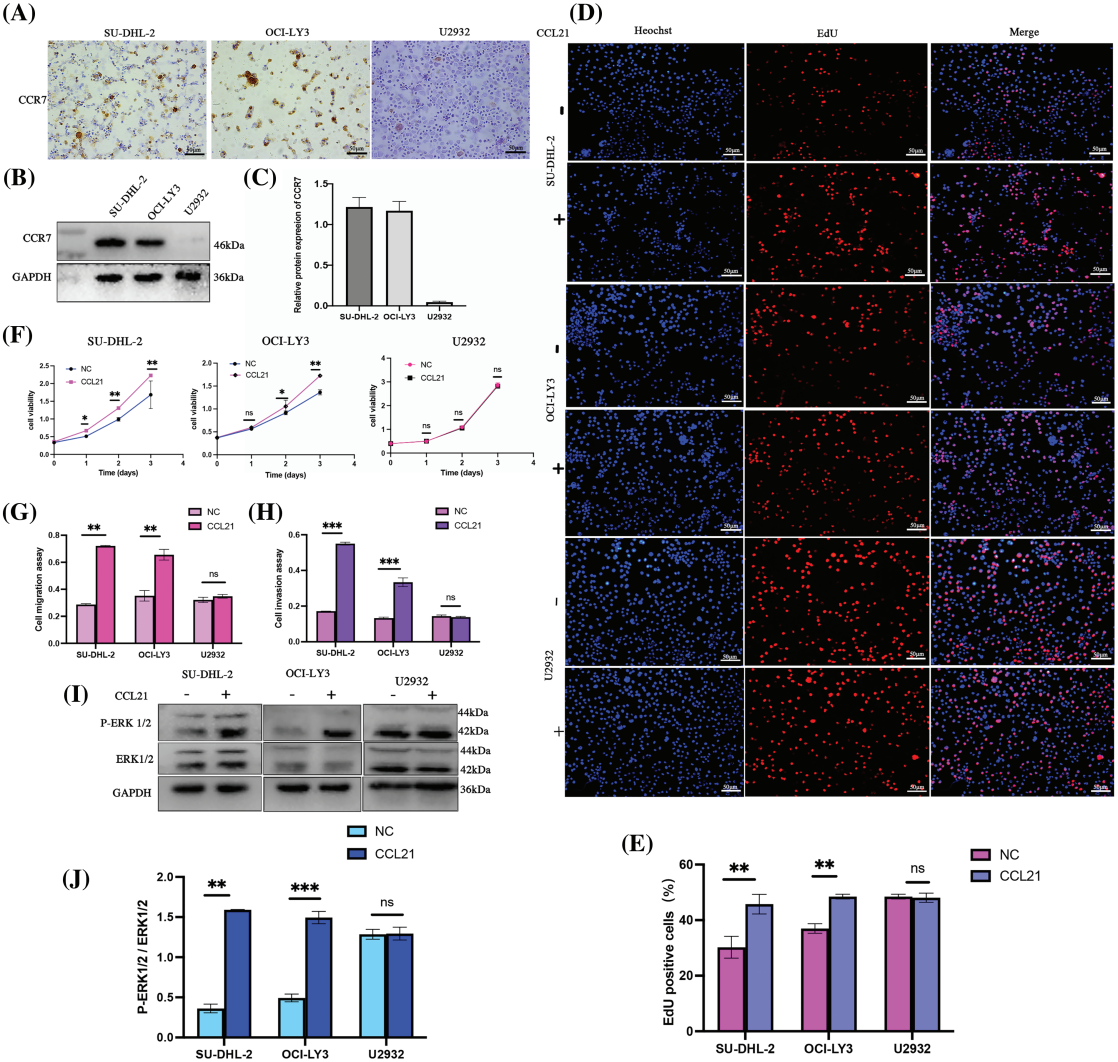
Activation of the ERK1/2 pathway by CCL21 through CCR7 enhances proliferation, migration, and invasion in DLBCL cells. (A–C) Analysis of CCR7 expression in OCI-LY3, SU-DHL-2, and U2932 DLBCL cell lines using IHC (×200) (A) and WB (B and C). (D–F) The impact of CCL21 on cell proliferation was assessed via EdU staining (D–E) and CCK-8 assays (F) across the three cell lines. (G and H) Transwell experiments demonstrated the effect of CCL21 on cell migration (G) and invasion capabilities (H) in the cell lines. (I–J) Analysis of the activation of the ERK1/2 signaling pathway by CCL21 (I) and its quantitative assessment (J) (^ns^*p* > 0.05, *p < 0.05, **p < 0.01, ***p < 0.001).

To elucidate whether the role of CCL21 in DLBCL is dependent on CCR7, our study delved deeper into their interplay. Various DLBCL cell lines (OCI-ly3, SU-DHL-2, and U2932) were selected, and CCR7 expression was analyzed using IHC (Fig. 4A) and WB (Fig. 4B). The results highlighted significant variability in CCR7 expression across these cell lines, with the SU-DHL-2 line showing the most pronounced expression and almost no expression detected in the U2932 line (Fig. 4C). The impact of human recombinant CCL21 protein (1 μg/mL) on cell proliferation was assessed using the CCK8 assay and EdU staining, revealing that CCL21 significantly promoted proliferation in OCI-LY3 and SU-DHL-2 cells (Figs. 4D–4F). Further, CCL21 significantly enhanced the migration and invasion capabilities of OCI-LY3 and SU-DHL-2 cells (Figs. 4G and 4H). To understand the effects of CCL21 binding to CCR7 on the ERK1/2 signaling pathway, the phosphorylation and total levels of ERK1/2 were analyzed through immunoblotting. The addition of CCL21 markedly activated ERK1/2 signaling in OCI-LY3 and SU-DHL-2 cells. In contrast, such activation was not observed in the U2932 cell line (Figs. 4I and 4J). These findings suggest that CCL21 may exert its oncogenic effects in DLBCL through CCR7, thereby playing a pivotal role in the progression of DLBCL.

### Lenalidomide may exert anticancer effects in DLBCL via CCL21/CCR7/ERK1/2 axis in vitro

**Figure 5:**
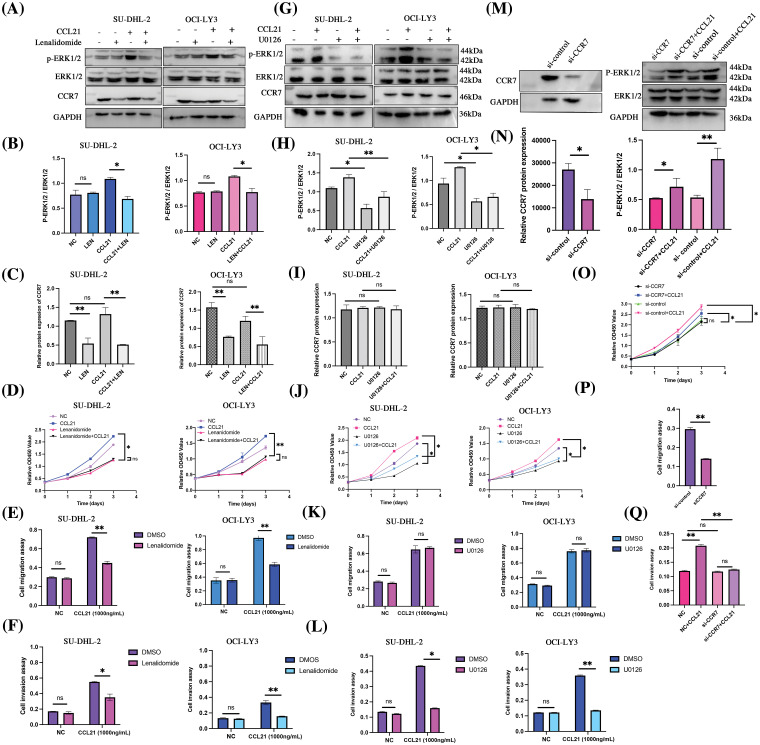
Lenalidomide modulates the CCL21/CCR7/ERK axis to suppress malignant biological behaviors in DLBCL cells. (A–C) Impact of lenalidomide and CCL21 on ERK1/2 phosphorylation and CCR7 expression. (D) CCK-8 assay evaluates the effect of lenalidomide and CCL21 on DLBCL cell viability. (E and F) Transwell migration (E) and invasion (F) assays demonstrate the regulation of cell behavior by lenalidomide and CCL21. (G–I) Influence of U0126 and CCL21 on ERK1/2 phosphorylation and CCR7 expression. (J) CCK-8 determination of the impact of U0126 and CCL21 on cell viability. (K and L) Effects of U0126 and CCL21 in transwell migration (K) and invasion (L) assays. (M and N) Analysis of ERK1/2 activity changes following CCR7 knockdown with CCR7-siRNA. (O–Q) Assessment of CCL21’s impact on cell viability (O), migration (P), and invasion (Q) after siRNA treatment (^ns^*p* > 0.05, *p < 0.05, **p < 0.01).

To elucidate the impact of lenalidomide on the CCR7/ERK1/2 axis, we initially treated SU-DHL-2 and OCI-LY3 cells with lenalidomide (90 and 70 µM, respectively), CCL21 (1 µg/mL), or a combination of both for 24 h. The lenalidomide inhibited the ERK1/2 activation induced by CCL21 (Figs. 5A–5C). Moreover, lenalidomide significantly inhibited the proliferation of SU-DHL-2 and OCI-LY3 cells stimulated by CCL21 (Fig. 5D), attenuated cell migration (Fig. 5E), and the enhanced invasion induced by CCL21 (Fig. 5F). Additionally, after 24 h of treatment with the MEK inhibitor U0126 (10 µM), analyses revealed that inhibiting ERK signaling did not affect CCR7 expression but blocked ERK1/2 activation, reversing the proliferation and invasion induced by CCL21 (Figs. 5G–5I). Transwell migration assays further demonstrated that U0126 inhibited the migration of cells enhanced by CCL21 (Fig. 5K), and the invasion ability was similarly diminished by U0126 (Fig. 5L). In the presence of CCL21, the siRNA-mediated knockdown of CCR7 significantly reduced the activation of ERK1/2 (Figs. 5M–5Q). These findings suggest that lenalidomide effectively inhibits DLBCL cell proliferation and invasion by modulating the CCL21/CCR7/ERK1/2 axis, highlighting its potential as a therapeutic target in DLBCL.

### Lenalidomide may exert anticancer effects in DLBCL via CCR7/ERK1/2 axis in vivo

**Figure 6:**
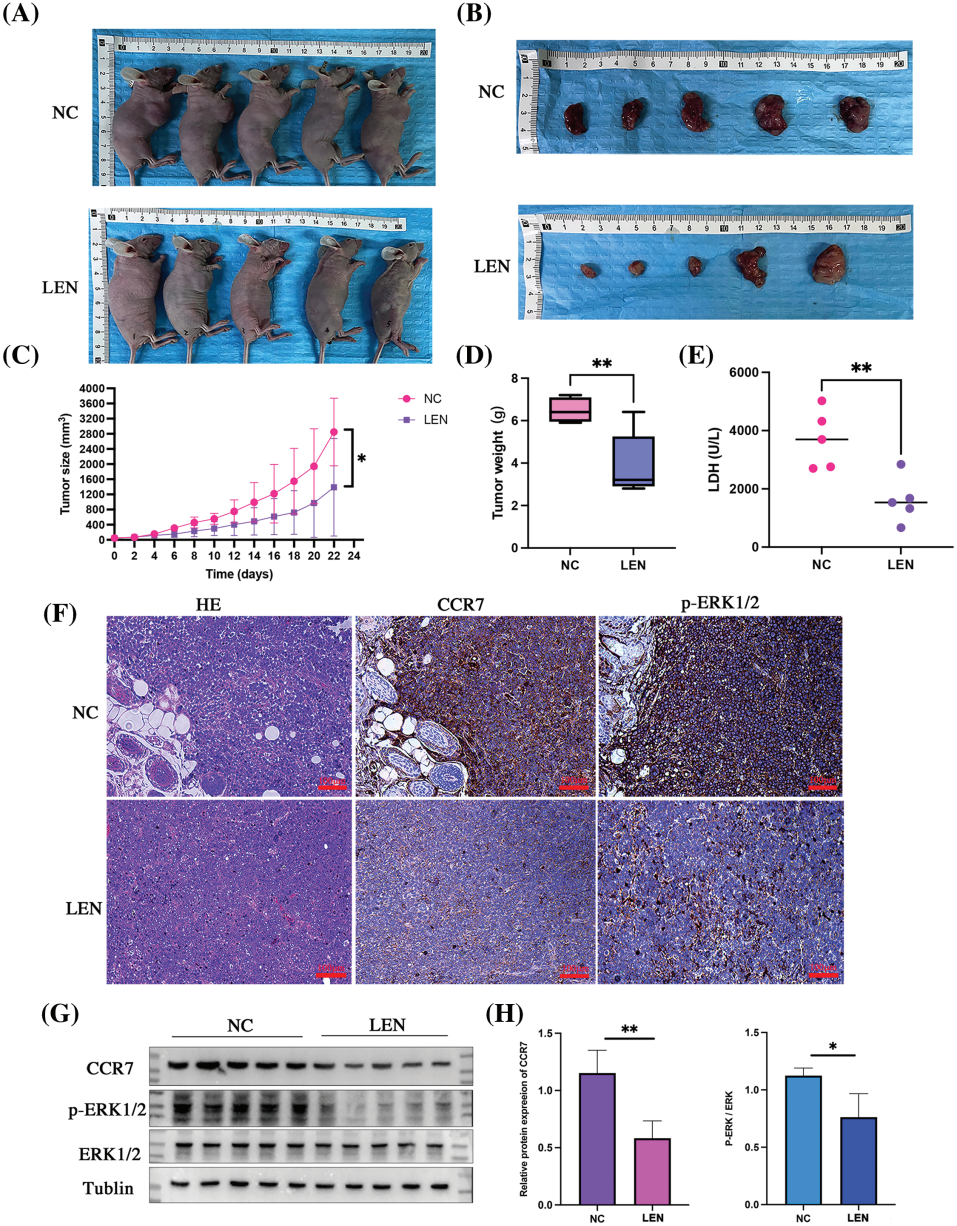
*In vivo* antitumor effects and mechanism of lenalidomide. Representative images of euthanized mice bearing tumors (A) and excised tumors from both groups (B). (C) Tumor growth curves following treatment with Lenalidomide and DMSO. (D) Tumor weights of mice after euthanasia. (E) Serum LDH levels in both groups. (F) HE and IHC staining showing CCR7 and p-ERK expression in tumor tissues from both groups (×200). (G and H) Protein expression of CCR7 and p-ERK in both groups (*p < 0.05, **p < 0.01).

To further explore the capability of lenalidomide in suppressing DLBCL tumor growth via the CCR7/ERK1/2 axis *in vivo*, we conducted animal experiments. The results demonstrated that the average tumor volume in the lenalidomide-treated group was significantly smaller than that in the control group (Figs. 6A and 6B). Fig. 6C reveals that the tumor growth curve in the lenalidomide-treated group significantly decelerated compared to the vehicle group (*p* < 0.05). Additionally, the mean tumor weight in the lenalidomide group was also lighter than in the control group (*p* < 0.05) (Fig. 6D). Serum total LDH levels in the lenalidomide group were lower than those in the control group (Fig. 6E). As illustrated in Figs. 6F and 6G, the expression of CCR7 and p-ERK1/2 significantly decreased in the lenalidomide-treated group. These encouraging *in vivo* results further clarify the potent role of the CCR7 /ERK1/2 axis in the mechanism of lenalidomide treatment for DLBCL.

## Discussion

Chemokines and their receptors play pivotal roles in the progression of various cancers [18]. Studies in B-cell malignancies have revealed subtype-specific patterns of chemokine receptor expression, underscoring their crucial function in the dissemination of malignant B-cells, leading to the formation of monoclonal B-cells and tumor transformation [19,20]. Specifically, in DLBCL, research has highlighted the importance of chemokine receptors. For instance, CCR7 has been identified as a B-cell homeostatic chemokine receptor [21] and an independent prognostic marker in DLBCL patients [11], while studies by Uhl et al. [8] have shown no significant correlation between other chemokine receptors and patient survival, aside from CCR7. Inspired by these findings, and considering the significant role of bioinformatics in cancer research [22–24], our study leverages biological information to identify CCR7 as a potential key gene in the action of lenalidomide in DLBCL. Furthermore, we validate the critical role of CCR7 in the prognosis of DLBCL patients and identify ERK1/2 as another independent prognostic factor. Through cellular and animal experiments, we explored the oncogenic role of the CCR7-CCL21/ERK1/2 axis in DLBCL. Given the significant role of lenalidomide in DLBCL treatment, our research specifically examined the impact of CCR7 on the response to lenalidomide treatment. Our findings suggest that lenalidomide may exert anticancer effects in DLBCL through modulation of the CCR7-CCL21/ERK1/2 signaling axis. Overall, our study not only underscores the importance of CCR7 in the prognostic evaluation of DLBCL patients but also provides a new target for developing treatment strategies against lenalidomide resistance.

Previous studies have demonstrated that CCR7 is activated upon binding to its ligands CCL19 and CCL21, initiating a cascade of cellular responses. This binding triggers G-protein activation, initiates the ERK1/2 signaling pathway, mobilizes calcium, and stimulates cell migration [25]. Similarly, the CCR3-CCL11 axis, specific to anaplastic large-cell lymphoma, enhances cell survival and promotes lymphoma cell proliferation through the activation of the ERK1/2 pathway [26,27]. Moreover, CCL28 has been shown to drive the growth and metastatic spread of breast cancer by activating the MAPK/ERK1/2 pathway, while the CXCL12-CXCR4 axis supports cancer cell proliferation, relying on the MAPK pathway for its effects [28]. The ERK1/2 pathway is pivotal in regulating cell proliferation and survival, with its abnormal activation linked to various cancer cell behaviors, including survival, proliferation, migration, and differentiation [29,30]. Inhibiting ERK1/2 has been found to trigger apoptosis in human DLBCL cells both *in vitro* and *in vivo* [31]. Recent findings also indicate a notable increase in p-ERK1/2 expression in recurrent/refractory DLBCL tumors compared to their initial diagnoses [32]. In our study, we established a clinical link between p-ERK1/2 expression and poor prognosis in DLBCL, and identified a significant positive correlation between CCR7 and p-ERK1/2 expression in DLBCL tissue samples.

Redondo-Muñoz et al. [33] initially discovered that the migration of B-cell chronic lymphocytic leukemia cells, induced by CCL21, could be inhibited by using anti-CCR7 antibodies, CCR7 siRNA transfection, or ERK inhibitors. Our findings are in harmony with these observations. Our *in vitro* experiments revealed that exogenous CCL21 administration elevated p-ERK1/2 expression, promoting tumor proliferation and invasion. However, this ERK1/2 activation enhancement was mitigated when CCR7 was silenced using small-interfering RNA or when treated with lenalidomide. Moreover, the proliferation and invasion capabilities heightened by CCL21 were partially reversed.

Lenalidomide is utilized at various stages of multiple myeloma (MM) management. Its antineoplastic effects include direct tumor cell proliferation and angiogenesis inhibition, and indirect modulation of immune cell populations within the tumor microenvironment, stimulating cytotoxicity. Its combination with R-CHOP as a front-line treatment in DLBCL patients has been shown to be safe by the Mayo Clinic [34]. Subsequent Phases I and II clinical trials demonstrated higher complete response rates and better progression-free survival in groups treated with lenalidomide compared to those without [35]. Nevertheless, specific patient populations that would benefit from lenalidomide remain to be confirmed. Given DLBCL’s heterogeneity and the limitations of cell of origin (COO) in guiding lenalidomide administration [36], it is crucial to incorporate early prognostic biomarkers into treatment planning and prognosis evaluation. Our experiments showed that Lenalidomide effectively reduces CCR7 expression, inhibits ERK1/2 activation, and significantly curtails tumor growth.

While our study provides promising insights into the role of CCR7-CCL21/ERK1/2 axis in DLBCL and the potential therapeutic impact of lenalidomide, it is not without limitations. Firstly, the heterogeneity of DLBCL might affect the generalizability of our findings across all subtypes of the disease. Secondly, the *in vitro* and *in vivo* models used may not fully replicate the complex tumor microenvironment encountered in human DLBCL, potentially affecting the translational relevance of our results. Lastly, while we suggest lenalidomide as a promising therapeutic agent, further clinical trials are necessary to validate its efficacy and safety in a broader DLBCL patient population. These limitations highlight the need for cautious interpretation of our findings and underscore the importance of future studies to address these gaps.

## Conclusion

In summary, our research conclusively demonstrates that CCR7 and ERK1/2 serve as crucial prognostic markers in DLBCL, with their interaction via the CCL21 pathway playing a pivotal role in tumor progression by activating ERK1/2. Importantly, our findings illuminate the potential of lenalidomide as a targeted therapy to inhibit the CCR7-CCL21/ERK1/2 axis, offering a promising avenue to mitigate DLBCL progression. This study accomplishes several key objectives. Firstly, it deepens our comprehension of DLBCL’s molecular mechanisms. Secondly, it paves the way for new avenues in the development of targeted therapies. These advancements hold the potential to markedly enhance patient outcomes.

## Data Availability

The datasets generated during and/or analysed during the current study are available from the corresponding author on reasonable request.

## Appendix A

**TABLE A1:** The primer sequences

Gene	Primer-F	Primer-R
IL6	TGTGTGAAAGCAGCAAAGA	ACCAGGCAAGTCTCCTCA
IL13	AAGCCACCCAGCCTATG	CCAGTGCCAACAGGAGA
CCL2	TTTTCCCCTAGCTTTCCC	GCAATTTCCCCAAGTCTCT
IL1B	TGTGCTGAATGTGGACTCA	ACAAAAGGGCTGGGGAT
CCL20	GACATAGCCCAAGAACAGAAA	AAGTCCAGTGAGGCACAAA
CXCL11	TCCAAGAAGAGCAGCAAAG	GCACACAATATCACAGCCA
CXCL8	GTGCTGTGTTGAATTACGGA	TTGACTGTGGAGTTTTGGC
CSF3	TCCTGTCCTCCCATCCC	CTCATCCCAGTGCCCATT
IFNB1	CCAACAAGTGTCTCCTCCA	TATTCAAGCCTCCCATTCA
CXCL13	CTCGACATCTCTGCTTCTCA	TTGGACACATCTACACCTCAA
CX3CR1	GCTGGTTCTTACGATGGC	CCACAAGGTGACTGGGA
TNFSF13B	CAGAAAGGCAGAAAGGAGAA	AATAAGTGACCACAGGGAATG
CCL19	TGGCGCTCACACATTCA	GGTCACAGACAGGCAGCA
CCL8	GAGAGATGGGTCAGGGATT	GGAGGTTGGGGAAAATAAA
CCR7	GCGATGCGATGCTCTCT	AAAGTTGCGTGCCTGGA
GAPDH	CTTTTGCGTCGCCAG	TTGATGGCAATATCCAC

**TABLE A2:** The association of CCR7 expression with diverse clinicopathological parameters in DLBCL

Characteristics	CCR7		*p* value
	Positive [N (%)]	Negative [N (%)]	
Gender			0.433
Male	22 (34%)	40 (66%)	
Female	17 (28%)	43 (72%)	
Age			0.571
<60	20 (34%)	38 (66%)	
≥60	19 (30%)	45 (70%)	
Lugano stage			<0.001
I–II	6 (8%)	67 (92%)	
III–IV	33 (67%)	16 (33%)	
Extranodal involvements			<0.001
≤1	8 (10%)	69 (90%)	
>1	31 (69%)	14 (31%)	
LDH			<0.001
Normal	8 (15%)	47 (85%)	
High	31 (46%)	36 (53%)	
IPI			<0.001
Low (0–2)	8 (11%)	64 (89%)	
High (3–5)	31 (62%)	19 (38%)	
Cell of origin (COO)			<0.05
ABC	33 (39%)	52 (61%)	
GCB	6 (16%)	31 (84%)	

**TABLE A3:** The association of p-ERK1/2 expression with diverse clinicopathological parameters in DLBCL

Characteristics	p-ERK1/2		*p* value
	Positive [N (%)]	Negative [N (%)]	
Gender			0.955
Male	22 (35%)	40 (65%)	
Female	21 (35%)	39 (65%)	
Age (years)			
<60	19 (33%)	39 (67%)	0.593
≥60	24 (37%)	40 (63%)	
Lugano stage			<0.001
I–II	13 (18%)	60 (82%)	
III–IV	30 (61%)	19 (39%)	
Extranodal involvements			<0.001
>1	26 (58%)	19 (42%)	
≤1	17 (22%)	60 (78%)	
LDH			<0.001
Normal	11 (20%)	44 (80%)	
High	32 (48%)	35 (52%)	
IPI			<0.001
Low (0–2)	15 (21%)	57 (79%)	
High (3–5)	28 (56%)	22 (44%)	
Cell of origin (COO)			0.210
ABC	33 (39%)	52 (61%)	
GCB	10 (27%)	27 (73%)	
